# Society Meetings

**Published:** 1902-10

**Authors:** 


### SOCIETY MEETINGS.

The American Association of Obstetricians and Gynecologists held its fifteenth annual meeting at Washington, D. C., September 16, 17, and 18, 1902. The Raleigh, which was the headquarters and in which the meeting was held, proved one of the most delightful of hostelries. To the already long list of interesting and instructive sessions held by the Association this one must be added, and among which it readily takes front rank. The weather was everything that could be desired—pleasant but not hot—and the attendance of Fellows was large and enthusiastic.

The scientific work of the association was well up to its high standard and the discussions, as usual, were crisp and animated. General George M. Sternberg, surgeon-general U. S. army (retired) delivered an address of welcome, to which President Ricketts made appropriate response; then followed the reading of papers and debates thereon, which continued during the next three days.

A reception was tendered President Ricketts on Tuesday evening by Dr. I. S. Stone, of Washington, to which the Fellows of the Association and their guests were invited, and which was enjoyed greatly by all present.

On the evening of Wednesday, the second day, the annual dinner was served in the lower banquet room of the Raleigh Hotel, at which upwards of fifty covers were laid. The decorations, music and service were of the very best. Several guests from New York and other cities attended the dinner as well as the scientific sessions, among whom were two from S. Paulo, Brazil, in the persons of Dr. Horace M. Lane and Dr. Guilherme Ellis. Among the guests from New York were Drs. Frank P.

Foster, editor of the *New York Medical Journal*, Dr. I. N. Love, editor of the *Medical Mirror*, Dr. B. T. Whitmore and William James Evans, Esq. From other cities came Dr. O. M. Willis, Marietta, and Dr. B. F. Beebe, Cincinnati, O.; Dr. R. G. Miles, New Castle, Pa.; Dr. E. E. Guenther, Newark, N. J.; Drs. Wm. A. Rolfe and H. H. Hartung, of Boston; Dr. Charles E. Congdon, Buffalo; Dr. Floyd J. Gregory, Keysville, Va.; Dr. Thomas J. McGuire, Parkersburg, W. Va.; Dr. Joseph A. Mudd, Hyattsville, Md., with a large attendance of the Washington profession, including Drs. Joseph Taber Johnson, J. Wesley Bovee, Isaac S. Stone, Thomas E. McArdle and others.

The officers elected for the ensuing year are as follows: president, L. H. Dunning, Indianapolis; vice-presidents, M. Rosenwasser, Cleveland and H. E. Hayd, Buffalo; secretary, William Warren Potter, Buffalo; treasurer, X. O. Werder, Pittsburg. Executive council: Edward Joseph Ill, Newark; William Henry Humiston, Cleveland; Edwin Ricketts, Cincinnati; Walter B. Chase, New York; A. Vander Veer, Albany, and L. S. McMurtry, Louisville. The next meeting will be held at Chicago, beginning the last Tuesday in September, 1903.

---

THE Roswell Park Medical Club has elected officers for the ensuing year as follows: president, Dr. Julius H. Potter; vice-president, Dr. William Meisberger; secretary and treasurer, Dr. George F. Cott.

---

THE Medical Society of the State of New York will hold its ninety-seventh annual meeting at Albany, January 27, 28 and 29, 1903. The president, Dr. Henry Reed Hopkins, of Buffalo, has appointed the following-named business committee: Dr. Ernest Wende, chairman, 471 Delaware Avenue, Buffalo; Dr. Hamilton D. Wey, Elmira, and Dr. J. Mongtomery Mosher, Albany. This committee will have charge of the program and all communications relating thereto should be addressed to one of the members.

---

THE Tri-State Medical Society of Alabama, Georgia and Tennessee will hold its fourteenth annual meeting at Birmingham, Ala., Tuesday, Wednesday and Thursday, October 7, 8 and 9, 1902, under the presidency of Dr. J. C. LeGrand, of Birmingham. A most interesting program has been prepared.

The Buffalo Academy of Medicine held meetings during the month of September as follows:

*Section on Surgery.*—Tuesday evening, September 2. Program: Tic douloureux and its surgical treatment, Wm. C. Phelps. Presentation of case by Marcel Hartwig; discussion by W. C. Krauss and others.

*Section on Medicine.*—Tuesday evening, September 9. Program: Syphilis of nervous system, Floyd S. Crego. Cerebral hemorrhage following injury to the spine, Sydney A. Dunham.

*Section on Pathology.*—Tuesday evening, September 16. Program: On the nature and germicidal action of the organic peroxides, F. G. Novy, Professor of Hygiene, University of Michigan, Ann Arbor, Mich. Statistics of movable kidney with special reference to nervous symptoms, A. L. Benedict.

*Section on Obstetrics and Gynecology.*—Tuesday evening, September 23. Program: Movable kidney; frequency, method of diagnosis and treatment, A. L. Benedict. Report of cases, (*a*) Tetanus following delivery; (*b*) Rupture of the uterus, Irving W. Potter.

*Section on Ophthalmology, Otology and Laryngology.*—Tuesday evening, September 30. Program: Adenoids, Carl Leo-Wolf, Niagara Falls. Some of the more common forms of ocular syphilis, Arthur G. Bennett. Presentation of cases of sinus thrombosis, Geo. A. Sloan. Radical antral operation, and partial extirpation of the larynx. Exhibition of patients, Geo. F. Cott.

